# A pilot investigation of audiovisual processing and multisensory integration in patients with inherited retinal dystrophies

**DOI:** 10.1186/s12886-017-0640-y

**Published:** 2017-12-07

**Authors:** Mark H. Myers, Alessandro Iannaccone, Gavin M. Bidelman

**Affiliations:** 1 Department of Anatomy and Neurobiology, University of Tennessee Health Sciences Center, Memphis, TN 38163 USA; 2 Department of Ophthalmology, Center for Retinal Degenerations and Ophthalmic Genetic Diseases, Duke University School of Medicine, Durham, NC USA; 3 School of Communication Sciences & Disorders, University of Memphis, Memphis, TN USA; 4 Institute for Intelligent Systems, University of Memphis, Memphis, TN USA

**Keywords:** Power spectral density analysis, Electroencephalography, Inherited retinal dystrophies, Event-related brain potentials

## Abstract

**Background:**

In this study, we examined audiovisual (AV) processing in normal and visually impaired individuals who exhibit partial loss of vision due to inherited retinal dystrophies (IRDs).

**Methods:**

Two groups were analyzed for this pilot study: Group 1 was composed of IRD participants: two with autosomal dominant retinitis pigmentosa (RP), two with autosomal recessive cone-rod dystrophy (CORD), and two with the related complex disorder, Bardet-Biedl syndrome (BBS); Group 2 was composed of 15 non-IRD participants (controls). Audiovisual looming and receding stimuli (conveying perceptual motion) were used to assess the cortical processing and integration of unimodal (A or V) and multimodal (AV) sensory cues. Electroencephalography (EEG) was used to simultaneously resolve the temporal and spatial characteristics of AV processing and assess differences in neural responses between groups. Measurement of AV integration was accomplished via quantification of the EEG’s spectral power and event-related brain potentials (ERPs).

**Results:**

Results show that IRD individuals exhibit reduced AV integration for concurrent audio and visual (AV) stimuli but increased brain activity during the unimodal A (but not V) presentation. This was corroborated in behavioral responses, where IRD patients showed slower and less accurate judgments of AV and V stimuli but more accurate responses in the A-alone condition.

**Conclusions:**

Collectively, our findings imply a neural compensation from auditory sensory brain areas due to visual deprivation.

**Electronic supplementary material:**

The online version of this article (10.1186/s12886-017-0640-y) contains supplementary material, which is available to authorized users.

## Background

Investigation of multi-modal stimulation has found that when auditory (A) and visual (V) stimuli are presented simultaneously, congruent information can change an individual’s object perception. If the dual stimulation is staggered in such a way as to convey a delayed time to contact of approaching stimuli, visual stimulation usually provides a more salient response. The timing of multisensory input may thus alter sensory integration in determining behavior and shaping perception [8]. For instance, two stimuli (e.g., concurrent AV stimuli) initiated to disparate sensory areas can result in a facilitation of enhanced sensory-perceptual processing [18].

Human neuroimaging studies reveal several underlying brain mechanisms responsible for AV processing [5]. AV stimuli elicit a complex cortical network featuring activation in the primary auditory and visual cortices, as well as several multisensory areas (superior temporal sulcus, intraparietal sulcus, insula, and pre-central cortex). However, several other studies have also found that unimodal sensory input can influence neural responses found in other distal areas normally responsible for processing different sensory modalities [30]. As an example, lip-reading from visual-only videos of mouth movement is associated with responses in auditory cortices (i.e., Heschl’s gyrus and Planum temporale) even if no auditory input is available [6, 24, 37]. These studies illustrate a dynamic interplay and cross-talk between uni- and multi-modal brain areas during AV processing.

The investigation of looming (approaching) and receding (fading) signals is a particularly promising avenue to address synergy between principles of multisensory processing. Looming signals dynamically increase in their effectiveness and spatial coverage relative to receding stimuli. It is also noteworthy that looming cues can indicate both potential threats/collisions and success in acquiring sought-after objects/goals [19, 21, 43], suggesting they are of high behavioral relevance.

In this study, we focused on AV integration in cases of inherited retinal dystrophies (IRDs), in order to further understand the neural mechanisms of multi-modal processing. IRD is a group of degenerative retinal diseases causing a progressive loss of photoreceptor cells. While the retinal micro-anatomical and functional visual characteristics of IRD have been and remain the object of intense investigation, its perceptual-cognitive consequences on AV processing remain largely unknown (cf. [1, 20]).

Several pieces of evidence suggest that IRDs might alter multisensory processing. Interestingly, IRD individuals often report that other sensory capabilities (e.g., hearing, taste) become more acute over time [1, 20]. The sensitization or marshalling of additional sensory areas has been known to compensate for singular sensory loss. For example, in individuals with early onset blindness, loud sounds can induce the illusory perception of flashes of light, suggesting an increase in visual awareness for certain auditory stimuli [1]. Such occurrences of putative ‘remapping’ or sensory ‘cross-talk’ may be the result of de-afferentation where sensory input to the auditory system begins innervating (or establishes stronger connections) to extra-striate visual areas. These cases reinforce the notion that connections in the adult brain can be modified when one sensory system becomes deficient and others are forced to provide compensatory processing.

In the current study, we aimed to characterize how the unimodal visual deficits of IRDs alter the perception and neurophysiological processing of AV stimuli, when the brain must bind (i.e., integrate) sound and visual cues. In this regard, IRD patients offer an ideal window for examining how visual deprivation might change sensory processing in a different modality (e.g., auditory) as well in tandem with the impaired visual sensory input (i.e., during AV processing). We show that IRD individuals have reduced AV processing and/or integration of multisensory cues, consistent with their impaired unimodal visual input, and those IRD patients demonstrate increased responsiveness to auditory stimuli, consistent with the notion that visual deficits are partially compensated by recruitment and perhaps expansion of auditory function [17]. We also assessed response identification accuracy (%) and reaction times (RTs) of the participants during AV processing to understand the behavioral implications of IRD’s putative cortical remapping on perception and motor control.

## Methods

Six patients (5 males, 1 female; *mean ± SD* age = 46.7 ± 23 yrs) with different types of IRDs were recruited in this pilot study: two with autosomal dominant (AD) retinitis pigmentosa (RP), two with autosomal recessive (AR) cone-rod dystrophy (CORD), and two with the related complex disorder, Bardet-Biedl syndrome (BBS) [15, 25–28]. Fifteen healthy non-IRD control participants (13 females, 2 males; age = 23.6 ± 2 yrs) were also recruited as baseline controls for this study. All participants had normal hearing by self-report. This study had the joint approval of the Institutional Review Boards of the University of Tennessee Health Science Center (IRB #: 13-02782-XP) and the University of Memphis (IRB #2370).

Diagnoses of the IRD patients was established via a combination of physician evaluation by a retinal degeneration expert (AI), psychophysical (Goldmann visual fields; dark- and light-adapted monochromatic automated perimetry), dark- and light-adapted flash electroretinogram (ERG), and imaging [spectral domain optical coherence tomography (SD-OCT) and fundus autofluorescence (FAF)] methods, and were further confirmed whenever possible (in 5 of the 6 participants) via molecular genetic diagnostic testing. The characteristics of the six patients are summarized in Table 1.

**Table 1:** IRD patient characteristics

	Age/Gender	Visual acuity	GVF size^a^	Flash ERG^b^	SD-OCT EZ/ONL^c^	Clinical diagnosis	Molecular diagnosis
PT1	50/M	20/200 OD, 20/125 OS	7 degs OU*	No rod or mixed measurable responses; only residual mixed ERGs recordable	Markedly generalized ONL/EZ drop-out with modest central residue	RP, autosomal dominant	*PRPF*31 C299R, heterozygous, known disease causing mutation
PT2	77/M	20/20 OU	10 degs OU	No rod or mixed measurable responses; only small flicker ERG recordable	Markedly generalized ONL/EZ drop-out with excellent central residue	RP, autosomal dominant	*RHO* P23H, heterozygous, known disease causing mutation
PT3	23/M	20/40 OU	Incomplete ring scotomas OU	Residual rod ERG; moderately reduced and electronegative mixed and cone ERGs	Mild generalized ONL thinning and ill-defined EZ across the whole scan	BBS, autosomal recessive	*BBS1* M390R, homozygous, known disease causing mutation
PT4	18/M	20/100 OD; 20/63 OS	Complete ring scotoma OU^d^	Non-recordable rod ERG; markedly reduced and electronegative mixed and cone ERGs	Mild generalized ONL thinning and ill-defined EZ across the whole scan	BBS, autosomal recessive	*BBS1* M390R, homozygous, known disease causing mutation
PT5	70/M	20/200 OU	Full OU, ill-defined central relative scotomas OU (no stimulus detection of targets I3e or smaller)	Delayed and mildly reduced rod and mixed responses, virtually non-recordable cone responses OU	Severe focal ONL/EZ loss centrally (partial sparing fovea/perifovea only)	CORD, simplex	Inconclusive, (*RPGRIP1L* G1105R), variant of uncertain significance (possibly pathogenic)
PT6	42/F	20/250 OU	Full OU, ~22-deg. size central relative scotomas to I4e target and ~15-deg. size central relative scotomas to III4e target OU (small foveal detection area in OS)	Normal rod responses; mildly reduced mixed a-waves; moderately reduced cone responses (flicker > transient) OU	Severe focal ONL/EZ loss centrally (partial sparing fovea/perifovea only, OS > OD) and underlying marked central RPE drop-out	CORD, simplex^e^	Testing in progress (via EyeGENE)

^a^GVF = Goldmann visual field, expressed as radius around fixation to a V4e target, in degrees (degs); when present (asterisk), thin far peripheral islands remote from central fields are not included in this calculation; ^b^ ERG = Electroretinogram; ^c^ SD-OCT = Spectral domain optical coherence tomography; EZ/ONL = ellipsoid zone/outer nuclear layer characteristics; RPE = retinal pigment epithelium

^d^Field of PT4 expanded greatly and reached its full size only after prolonged light adaptation, consistent with markedly delayed light adaptation as would be expected in a ciliopathy like BBS

^e^The diagnosis of CORD in PT6 is based not on ERG results, but on monochromatic automated perimetry evidence of markedly elevated/non-measurable rod-mediated thresholds (dark-adapted 500 nm stimuli) within the central 30 degs of visual field

IRD patients were selected for this study to be representative of three different scenarios (Additional file 1: Figure S1): (i) severe peripheral vision loss with concentric visual field constriction and central visual preservation as in classical RP, in which the primary cells affected by the disease are the rods (rod > cone disease; Additional file 1: Figure S1A-B); (ii) severe central vision loss with peripheral preservation as in CORD, in which there is a cone > rod disease pattern (Additional file 1: Figure S1C-D); and (iii) moderately severe vision loss across the board with moderate to fair preservation of both peripheral and central vision as in BBS patients, a ciliopathy in which both rods and cones are affected with marked shortening of the outer segment but, at least at the initial stages we chose, with only ring scotomas and fairly good preservation of both peripheral and central visual function (Additional file 1: Figure S1E-F). The objective was that of choosing patients representative of vision loss patterns to begin testing the hypothesis that all scenarios would affect the response to A, V and AV stimuli, but that they would differ in their abnormality patterns to looming vs. receding stimuli, with BBS patients predicted to be the closest to normal to both types of stimuli.

### Audiovisual stimuli and behavioral task

**Fig. 1:**
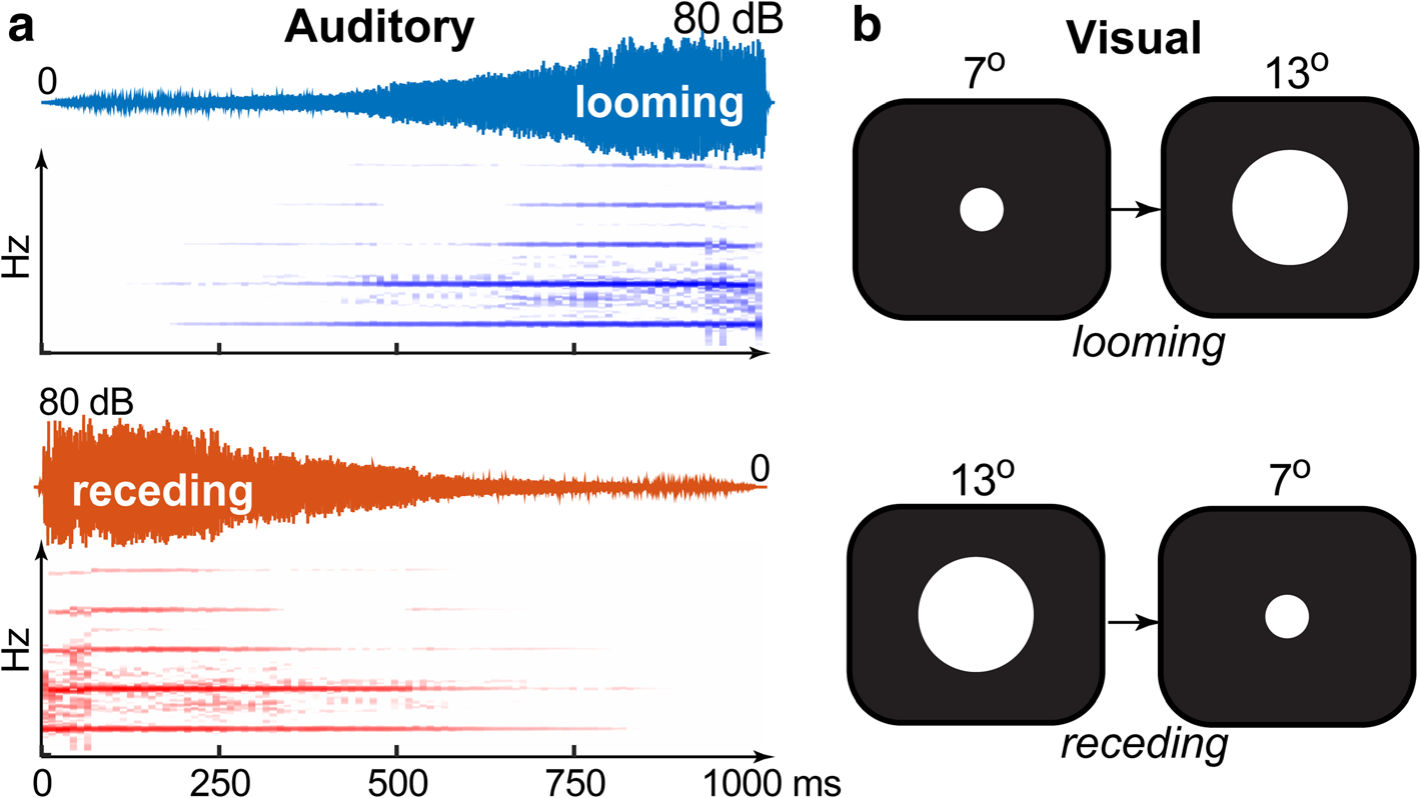
Stimuli and behavioral paradigm. Participants perform detection of moving (looming, receding) stimuli that could be **a** auditory, **b** visual, or multisensory auditory-visual. **a** Auditory time waveforms (top) and spectrograms (bottom). **b** Visual stimuli increased (looming) or decreased (receding) in size. Perception of movement was induced by linearly changing the size of the centrally displayed disk for the visual condition and by changing the intensity of a complex tone for the auditory condition over 1000 ms (e.g., [14])

The experiment involved the identification of stimuli that could be looming or receding auditory, visual, or multisensory audiovisual (A, V, and AV, respectively), as described in Cappe et al. [14]. To induce the perception of looming movement, visual stimuli (detailed below) changed in size and auditory stimuli changed in intensity by 80 dB over 1000 ms so as to give the impression of “looming” (i.e., approaching; 0 → 80 dB SPL) or “receding” (80 → 0 dB SPL) movement. In the present study, looming stimuli were treated as targets and receding stimuli as catch trials (not analyzed in this study). Each the three conditions were repeated 150 times across 15 blocks of randomly intermixed trials. On each trial, participants were asked to judge if the stimulus was “looming” or “receding” via a button press on the computer. They were encouraged to respond as accurately and as quickly as possible. Both response identification (%) and reaction times (RTs) were recorded. The stimulus conditions are schematized in Fig. 1.

#### Auditory stimuli

Sound tokens comprised 80 dB rising-intensity (looming signal) and falling intensity (receding signal) 1000 Hz complex tones composed of a triangular waveform. Auditory stimuli were generated with Adobe Audition software (Adobe Systems Inc.). Prior research has shown that these types of complex tonal stimuli produce more reliable looming and receding percepts [36] and may also be preferentially involved in multisensory integration compared to simple tones [32, 41]. Auditory stimuli were presented over Etymotic ER-2 insert earphones (Etymotic Research). Tokens were sampled at 48 kHz and were 1000 ms including 10 ms onset/offset ramps (to avoid audible clicks).

#### Visual stimuli

Visual tokens consisted of a centrally presented disc (white on a black background) that symmetrically expanded (from 7° to 13° diameter with the radius increasing linearly at a constant rate) in the case of looming stimuli or contracted (from 13° to 7° diameter) in the case of receding tokens. Visual tokens were presented on Dell 27″ U2713 LED computer monitor at a distance of 90 cm.

Multisensory AV stimuli featured the combined auditory and visual tokens described above. All stimuli were matched in total duration (1000 ms). The interstimulus interval was varied from 800 to 1400 ms (rectangular distribution) to avoid participants anticipating the timing of stimulus presentation. A focal point in the form of a fixation cross (+) was presented on screen between trials to control gaze and minimize saccades. Every participant was able to visualize and fixate foveally the fixation target. Stimulus delivery and response recording was controlled by custom routines coded in MATLAB® 2013 (The MathWorks, Inc).

### EEG recordings

Evoked brain responses were recorded using Neuroscan SynAmps RT amplifiers. A 64-channel sintered Ag/AgCl electrode array (QuikCap, Compumedics NeuroScan) was used to record neuroelectric activity from around the scalp using an average referenced montage. Additional electrodes placed on the outer canthi of the eyes and the superior and inferior orbit monitored ocular activity. Specific EEG hardware and recording parameters (presentation rate, filtering, sample rate, etc.) followed typical procedures from our laboratory [11, 12]. Electrode contact impedance was maintained <5 kΩ. Subjects reclined comfortably in an electroacoustically shielded IAC sound booth during testing. EEG signals were amplified, online filtered (0.1–500 Hz), and digitized at 1 kHz per channel. Analysis epoch windows for the cortical event related potentials (ERPs) spanned −100 to 1100 ms to encompass an appropriate pre-stimulus interval for baseline correction and the extent of the stimulus trial. Individual epochs contaminated by myogenic noise were manually discarded and blink artifacts (±50 μV) rejected prior to averaging using principal component (PCA) decomposition [46]. Preprocessing was conducted in the Curry 7 Neuroimaging Suite (Compumedics Neuroscan) and custom routines coded in MATLAB and EEGLAB [16].

### Power spectral density analysis

**Fig. 2:**
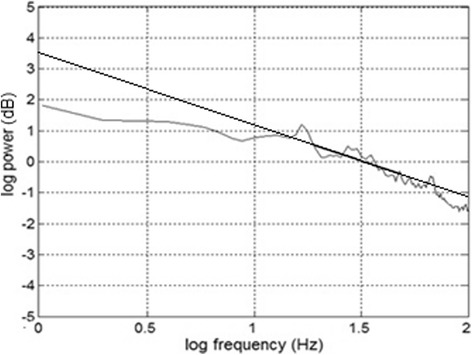
Linear regression analysis of PSD spectral slopes of the EEG spectrum of control participant

Power spectral density (PSD) analysis enables the characterization of neural activity with respect to its frequency distribution. PSDs were computed at each electrode location per subject and stimulus to quantify the amount of neural power (measured in μV) occurring at a given sensor location and across frequency. An example of the power spectral density measured at a representative electrode is shown in Fig. 2.

Linear regression was then performed across the frequency range in order to quantify the overall tilt of the PSD spectrum over the bandwidth from 1 to 100 Hz. A negative slope value (−α) typically conforms to the fractal dimensionality of 1/f^α^ (Fig. 2). This analysis enabled us to determine if there was a uniform distribution of power across the scalp EEG (slope = 0), or rather, an increase/decrease in lower- (alpha: 8-15 Hz) vs. higher-frequency (gamma: 20-80 Hz) bands of the EEG (slope ≠0). In particular, beta-gamma neural frequency activity has been associated with cognitive processing [7, 9, 10] and may be associated with congenital neuropathic conditions or trauma to areas of the cortex [33, 34]. PSD slopes were computed for each electrode location and participant. This resulted in a topographic map of PSD values across the scalp. We compared groups’ PSDs for each stimulus condition using a *t*-test on the topographic maps (threshold masked at *p* < 0.05). This approach identified regions of electrode clusters which distinguished AV responses in control and IRD listeners. Multiple comparisons were corrected via the Benjamini-Hochberg method to reduce the false discovery rate (FDR) [4].

### Statistical analysis

Two-way mixed model ANOVAs were used to analyze the behavioral data (%, identification and RTs) with factors group (IRD, control) and stimulus (A, V, AV). Subjects served as a random factor. The behavioral responses we aimed to measure were categorical binary judgments (i.e., “looming” vs. “receding”). Consequently, we used a binomial distribution and canonical logit link function in the ANOVA model for identification scores. RTs were analyzed using a similar ANOVA only with a normal Gaussian distribution to model the residuals, given the continuous nature of RT responses. Although age was not correlated with EEG measures (*r*_*s*_ = −0.18, *p* = 0.15), on average, IRD patients were older than controls (IRD: 46 ± 23 yrs.; controls: 23.6 ± 2 yrs). Consequently, we used age as covariate in the ANOVA models to partial out potential age-related changes in the evoked potentials ([13]a). An a priori significance level of ɑ = 0.05 was used for all statistical testing.

## Results

**Fig. 3:**
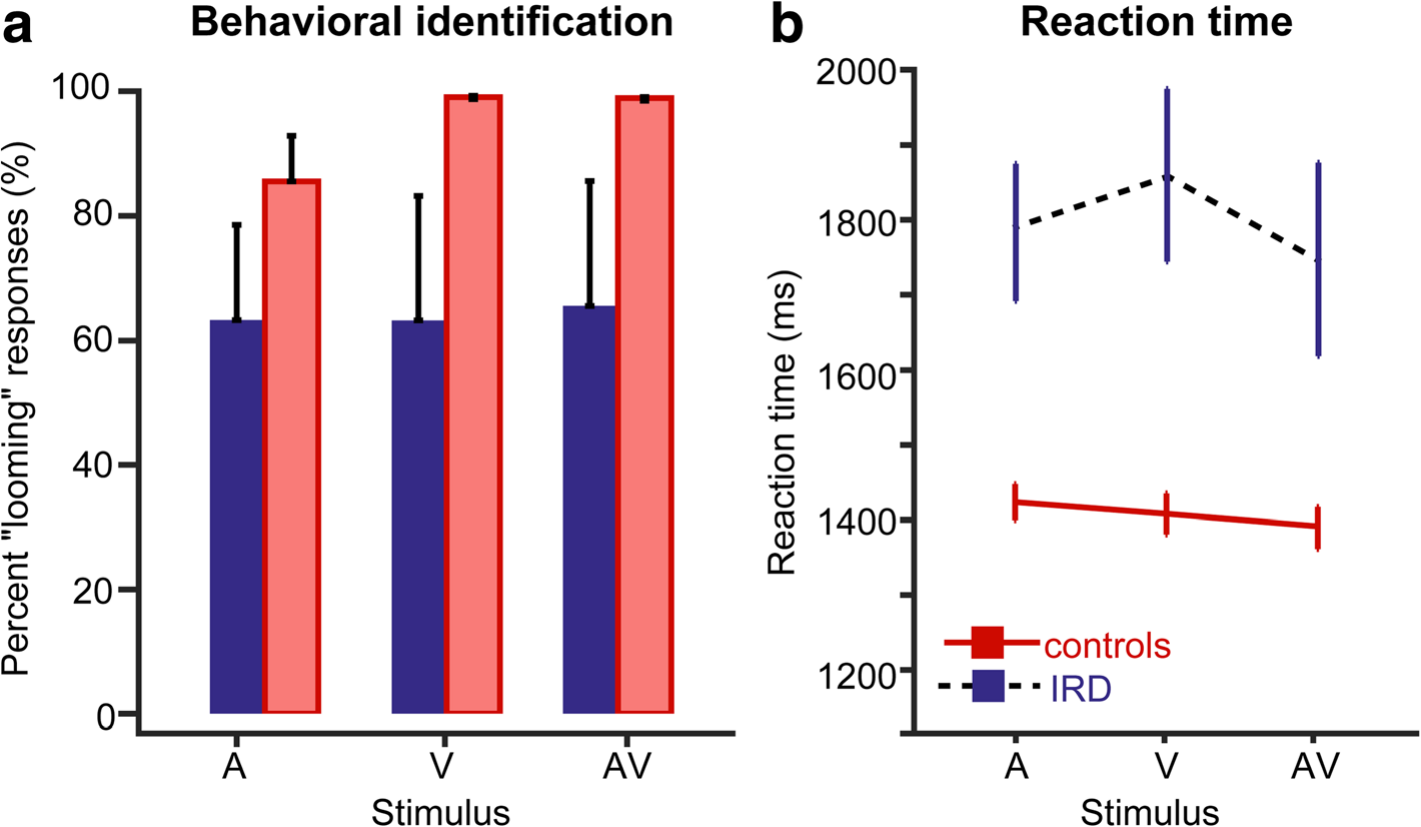
Grand average behavioral identification (**a**) and reaction times (**b**) for correctly judging “looming” audiovisual stimuli for the control and IRD group. Error bars = ±1 s.e.m

Behavioral identification and reaction times for correctly judging “looming” stimuli are shown in Fig. 3. An ANOVA (logistics model) conducted on identification judgments revealed a significant group x stimulus interaction [*F*_2,38_ = 86.32, *p* < 0.0001, ${\eta}_p^2$= 0.82], after accounting for age. Multiple comparisons indicated this interaction was attributable to group differences in the V (*p* = 0.0248) and AV (*p* = 0.027) conditions; no difference between groups was observed in the unimodal A condition (*p* = 0.93). That is, the propensity to correctly identify stimuli was better in the control relative to IRD group for V and AV tokens whereas group identification was similar for A tokens.

Analysis of the RTs similarly revealed a group x stimulus interaction [*F*_2,38_ = 3.52, *p* = 0.039,${\eta}_p^2$ = 0.22], after accounting for age. Post hoc comparisons revealed that controls were ~400 ms faster (1407 ± 93 ms) in judging audiovisual stimuli than IRD participants (1796 ± 269 ms) across the board [main effect of group: *F*_1,38_ = 27.71, *p* < 0.0001 ${\eta}_p^2$ = 0.42]. Yet, the interaction again suggests this group difference depended on the stimulus condition. Post hoc comparisons indicated that IRD patients were slower at responding than controls for each stimulus conditions (A, V, AV; all *p-*values <0.0001). Within the IRD group, patients showed slower RTs when judging unimodal V stimuli compared to multimodal AV stimuli (*p* = 0.006), consistent with their visual deficits. The comparison between RTs for the V and A stimuli in the IRD group was marginal (*p* = 0.07). In contrast, RTs were similar across stimuli for the control group (*p*s > 0.25), yet trending in the expected direction (fastest for the AV multimodal condition). Collectively, these analyses indicate group differences in the perceptual identification of both A and V stimuli that convey motion, with slower and less accurate judgments in IRD patients.

**Fig. 4:**
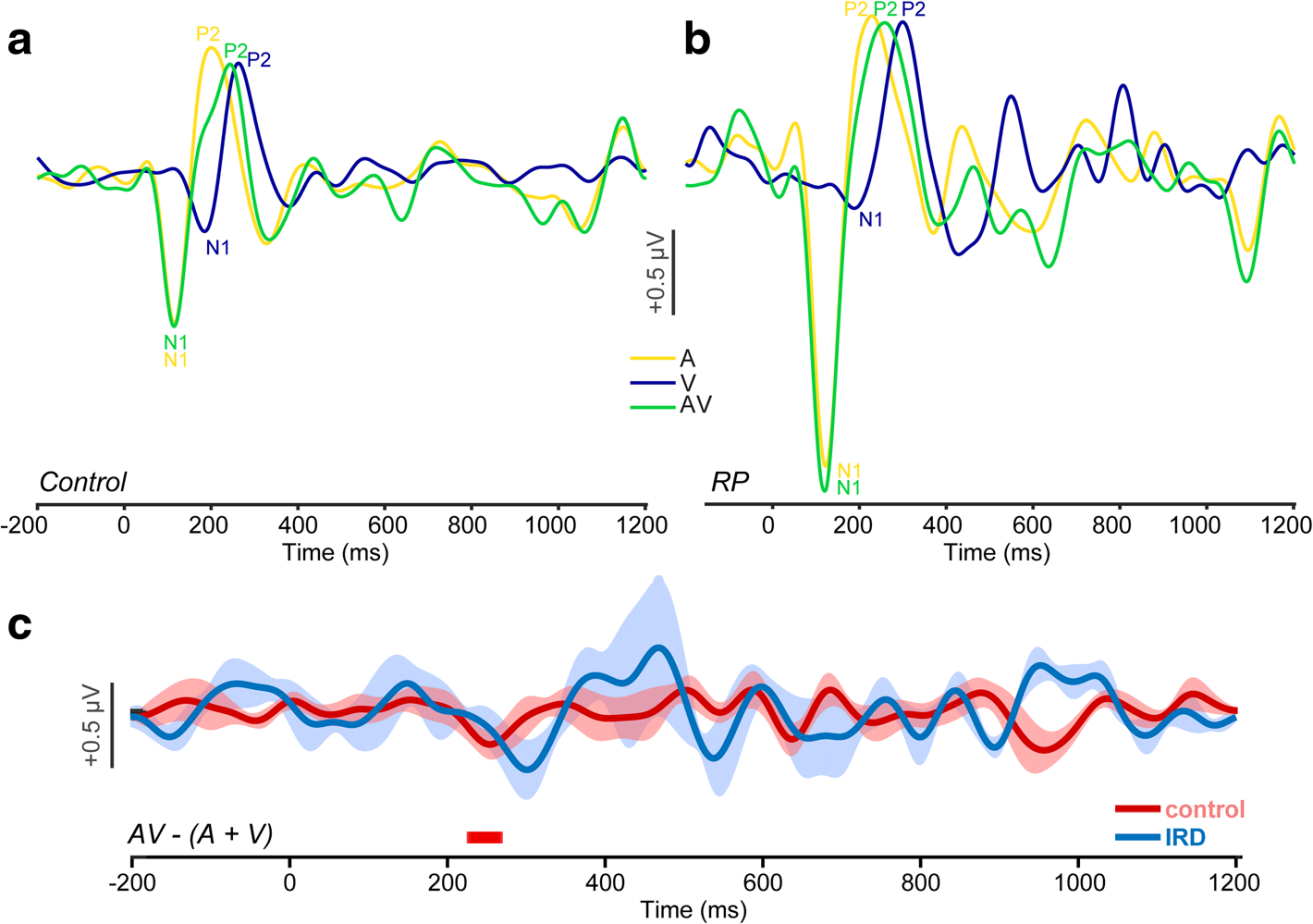
Cortical event-related potentials (ERPs) following audiovisual stimuli recorded at Cz. ERPs to A, V, and AV stimuli in controls (**a**) and IRD (**b**) patients. In both groups, unimodal auditory responses elicit the largest N1/P2 deflections, with later responses for the visual N1. Across tokens, IRD patients show larger cortical responses than controls (e.g., compare N1 magnitudes). **c** Group comparisons of the audiovisual integration effect (difference wave) contrasting uni- and multimodal ERPs [i.e., AV – (A + V)]. Potentials different from zero indicate an enhancement in AV processing compared to the summed unimodal (A + V) responses. Bars below the traces show time segments where AV > (A + V) (i.e., significant multisensory processing, *p* < 0.05). Despite larger unimodal responses in IRD patients, only the controls show significant AV integration (~200 ms) after initiation of the audiovisual stimulus. Shading = ± 1 s.e.m

Our initial analysis of possible group differences in multisensory neural encoding assessed time-locked ERP responses to A, V, and AV stimuli. ERPs to looming A, V, and AV stimuli are shown for control and IRD patients in Fig. 4. Generally speaking, IRD patients exhibited larger (i.e., more robust) negative N1 responses (see deflection at 100 ms) for A and AV stimulation, suggesting higher sensitivity to auditory stimuli.

To more directly quantify group differences in multisensory neural processing, we computed a difference waveform analysis between the combined AV and unimodal A and V conditions [i.e., AV - (A + V)]. This difference potential allowed us to examine the degree to which the multimodal AV stimulus produced a facilitation effect compared to the sum of the unimodal conditions alone [45]. Results of this analysis are shown in Fig. 4c. We used a sample-by-sample *t*-test contrasting these potentials against a zero baseline to assess significance of AV responses per group [10, 23]. That is, the combined AV ERP must be larger than the sum of the unimodal responses to confirm multisensory processing for a given group. We required that running significant periods persist for >20 ms to be considered reliable and help further control false positives (e.g., [23]). This analysis revealed significant multimodal processing at a latency of ~200 ms in the control cohort. The direction of this effect was negative, suggesting that the combined multimodal stimulus produced a slightly suppressed response compared to the sum of the individual constituents (i.e., coactivation). Despite the larger unimodal auditory responses (cf. Fig. 4a and b) seen in IRD patients, they did not show reliable AV enhancement. That is, IRD’s multimodal response to AV stimulation was more variable and not significantly different from the summed unimodal responses, suggesting no superadditive (cf. integration) of the two senses [45].

**Fig. 5:**
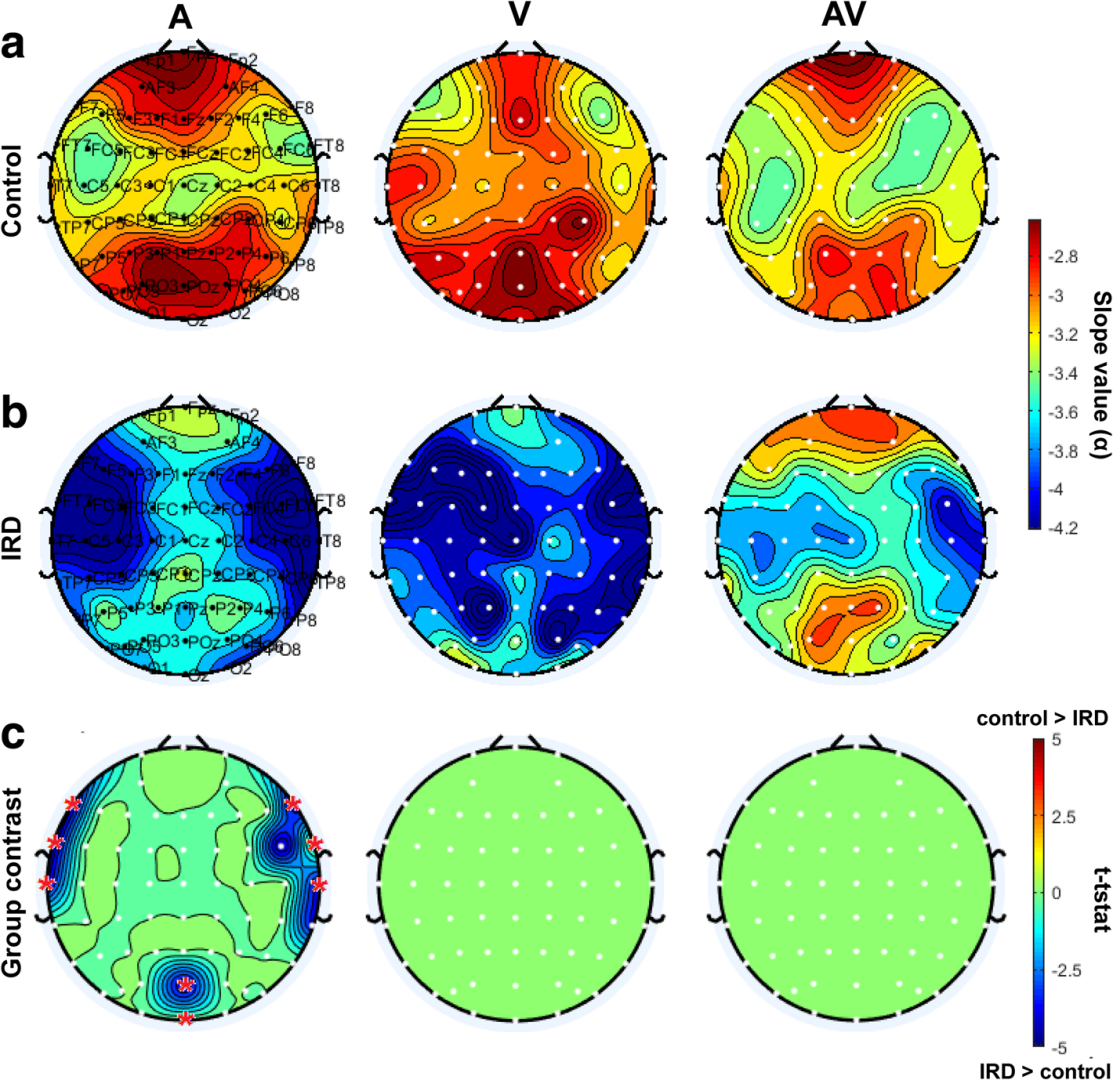
Topographic maps of PSD spectral slope values (see Fig. 2) in *control* (**a**) and *IRD* (**b**) participants for A, V and AV stimuli. **c** Topographic map contrasting IRD and controls (i.e., Fig. 5a vs. b) (*t*-test; FDR threshold masked at *p* < 0.05). Significant differences in the audio-looming condition were observed between groups at temporal and occipital electrode sites (* = F7, FT7, T7, F8, FT8, T8, POz, Oz). IRD patients show increased PSD compared to controls, consistent with their larger unimodal audio responses observed in the ERPs (e.g., Fig. 4). Cool colors (i.e., negative *t*-stat) indicate scalp locations where IRD slopes > controls

The scalp distributions of PSD slope values (α) (see Fig. 2) for the A, V and AV stimuli are shown for the control group in Fig. 5a. For controls, A stimulation produced smaller PSD slope values, particularly in frontal and occipital areas, indicating more uniform EEG spectral power in these regions. Similarly, V stimuli produced smaller PSD slopes over the occipital cortex. Lastly, AV stimuli produced smaller PSD slopes primarily over frontal brain areas.

Starkly different EEG spectral power distributions were observed among IRD subjects (Fig. 5b). A and V stimulus produced an increased (steeper) EEG PSD tilt, particularly over temporal regions (e.g., auditory cortex). Paralleling controls, AV stimuli produced lower PSD slopes in the frontal and occipital areas, although the effect was weaker.

We directly compared PSD topographic maps between groups using an independent samples *t*-test corrected for multiple electrode comparisons using FDR (Fig. 5c). Results of this initial analysis (after FDR correction) showed significant group differences (i.e., IRD > control) only in the unimodal A condition for electrode clusters over the bilateral temporal and occipital cortices (channels F7, FT7, T7, F8, FT8, T8, POz, Oz; see Fig. 7, red asterisks). IRD patients had larger, more negative PSD spectral slopes than controls in these areas consistent with the ERP results (see Fig. 4).

**Fig. 6:**
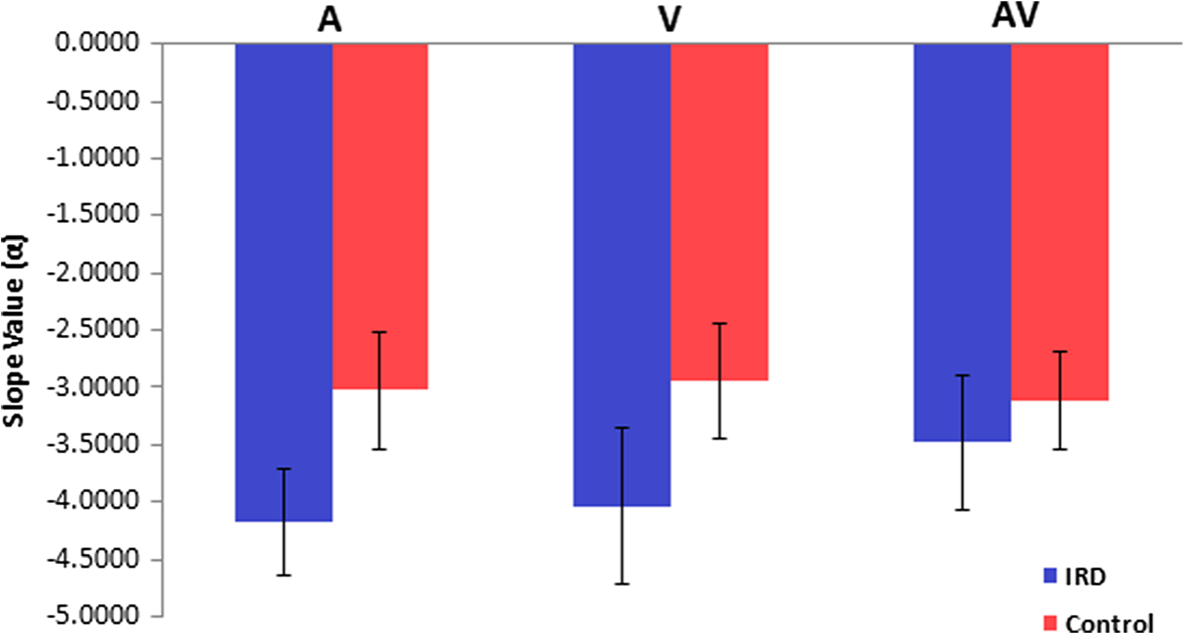
Comparison of PSD slopes between IRD and controls for A, V and AV stimuli. Slope values represent the average spectral slope pooled across eight electrodes (F7, FT7, T7, F8, FT8, T8, POz, Oz). Error bars = ±1SD

To quantify the effects of stimulus type (A, V, AV) and group (IRD, control) on EEG spectral power slopes, we averaged PSD slopes across electrodes showing prominent group effects in our topographic analysis (e.g., Fig. 5c: F7, FT7, T7, F8, FT8, T8, POz, Oz) (Fig. 6). An ANOVA conducted on PSD values revealed a group x stimulus interaction after controlling for age [*F*_*2*,33_ = 6.82, *p* = 0.0033 ${\eta}_p^2$ = 0.29]. By group, post hoc comparisons revealed that the slopes of controls did not differ across stimulus conditions (all *p* values >0.82). In contrast, the slopes of IRD subjects were larger (i.e., more negative) for V compared to AV stimuli (*p* = 0.0008). Comparisons by stimulus revealed that IRD patients exhibited larger PSD slopes in the unimodal auditory (A, *p =* 0.029) and visual (V, *p* < 0.001) conditions but similar responses for the multimodal AV stimulus (*p* = 0.65).

**Fig. 7:**
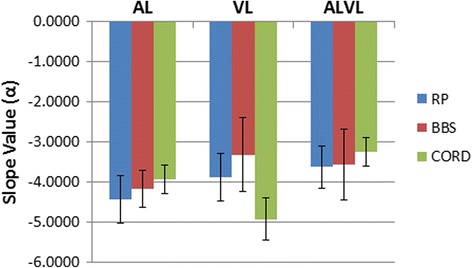
AV processing in RP participants with different pathologies (*n* = 2/group). Bardet-Biedl syndrome (BBS) subjects exhibit similar EEG PSD slopes within a similar range, and CORD participants exhibit very high PSD slope values during V stimulation

The IRD subgroups exhibited various neurological responses to the stimuli, seen in Fig. 7. RP participants appeared to have the highest average values in this group, corresponding to alpha activity across the cortex during AV stimuli. CORD participants produced very high PSD slopes in their EEG during V stimulation likely reflecting high theta-alpha activity, whereas, BBS subjects seemed to exhibit relatively normal beta-gamma activity. While these analyses suggest possible differences in AV processing between IRD pathologies, we note that the small sample size of each subgroup (*n* = 2) limits a quantitative comparison of the RP, BBS, and CORD stratifications.

**Fig. 8:**
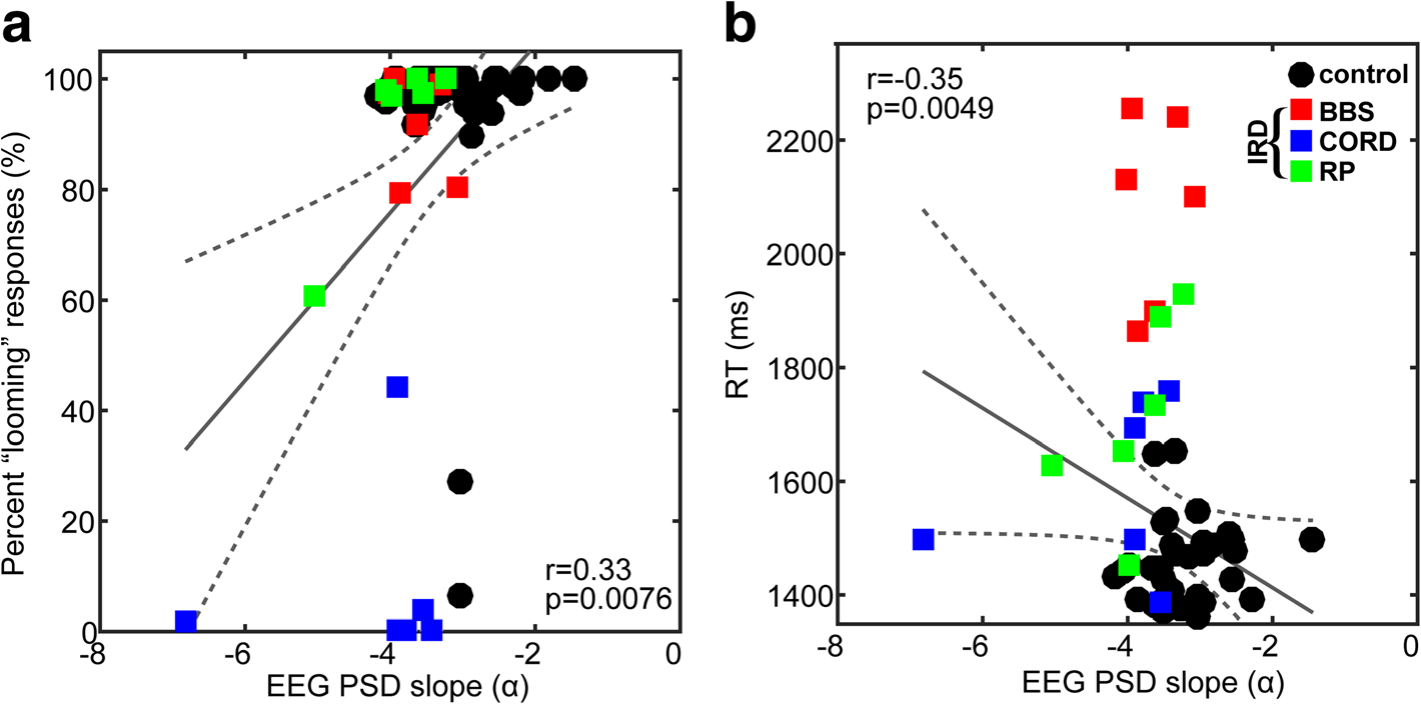
Brain-behavior relations between neural PSD slopes (i.e., EEG spectral tilt) and **a** behavioral identification (% “looming” responses) and **b** reaction times for identifying “looming” vs. “receding” motion in AV stimuli. Larger PSD slopes (i.e., less broadband EEG spectrum) indicative of IRD patients corresponds with poorer performance in identifying the perceived motion of AV stimuli. Similarly, more negative PSD slopes predict slower RTs in categorizing AV stimuli. The different etiologies of IRD (i.e., BBS, CORD, IRD) are shown as color coded symbols. Solid line, linear regression fit (MATLAB fitlm, bisquare weighting) to the pooled data (across all subjects); Dotted lines, 95% CI

Lastly, we assessed possible relations among individuals between behavioral (% identification accuracy for “looming” stimuli, RTs) and neural (PSD) responses via Spearman correlation analyses (Fig. 8). When considering all stimuli, EEG PSD slopes were positively associated with behavioral accuracy, such that shallower (more positive) spectral slopes predicted better behavioral identification (*r*_*s*_ = 0.33, *p* = 0.008) (Fig. 8a). In contrast, RTs were negatively associated with neural PSD slopes, such that that shallower (more positive) tilt of the EEG spectrum predicted faster behavioral decision times for judging AV stimuli (*r*_*s*_ = −0.35, *p* = 0.005) (Fig. 8b). Collectively, these findings help clarify the behavioral relevance of the neural PSD group effects: steeper, more negative spectral tilt of the EEG (characteristics of IRD subjects) is associated with less accurate and slower judgments of AV stimulus identity.

## Discussion

In the current study, we measured cortical ERPs and spectral power of the EEG in normal controls and individuals with long-term visual impairments (IRD patients) in response to uni- and multi-modal audiovisual stimuli. Collectively, results revealed reduced behavioral sensitivity and cortical responsiveness in IRD patients (relative to controls) to stimuli when visual cues were present (i.e., V and AV conditions). While controls showed significant multisensory neural integration between auditory and visual inputs, we did not observe this integrative processing in IRD patients. Yet, IRDs showed larger evoked potentials than healthy controls in response to stimuli containing sound cues (A, AV tokens), an auditory bias that was paralleled in their behavioral accuracy. This latter finding implies that the auditory system may help compensate multisensory signal processing following loss or impairment of the visual sensory modality.

Notably, IRD patients show enhanced ERPs for A and AV conditions but did not show AV integration per se, as was hypothesized from non-IRD studies or other degenerative eye diseases [29, 35]. We infer that as one sensory area (i.e., vision) declines in function over time in IRD subjects, other sensory areas (i.e., audition) are recruited to aid perceptual processing. Our findings are consistent with the notion that visual deficits due to permanent or progressive blindness are partially compensated by recruitment and/or expansion of the auditory system [17] rather than differential changes in integrative processing. Our data is also in line with studies in late-onset blindness which have shown cross modal sensory reorganization via electrophysiological recordings [39]. Visual cortical reorganization may involve normally-developed striate and extrastriate visual areas, which are presumably involved in visual imagery [40]. Late-onset blindness utilizes these formally developed cortical structures which may be activated through bidirectional auditory and visual sensory pathways. The occipital cortex can be activated through non-visual stimulus in blind subjects via auditory tonal stimuli, suggesting that primary visual areas may be not be as sensory specific as traditionally thought [40]. It is conceivable that, in cases of progressively degenerating vision (as in IRDs), the brain initiates sensory reorganization and compensates by recruiting multisensory neurons in both auditory and visual cortices. This may account for the larger responsiveness to stimuli containing auditory cues in IRD subjects relative to controls.

In this regard, our data are in agreement with fMRI studies which reveal an expansion of the tonotopic maps of auditory cortex in blind patients compared to sighted individuals [17]. These studies suggest that visually impaired individuals tend to exhibit greater reliance on other sensory inputs to maintain the same degree of perceptual discrimination. Our results demonstrate a similar entrainment of large cortical populations from auditory stimulation in IRD subjects. Due to the gradual loss of vision in IRDs, larger neural networks might be constructed via activation of previously dormant connections and/or emergence of new neural pathways between distant cortical regions.

In particular, our behavioral identification results (Fig. 3a) are consistent with the fact that, in IRD subjects like the ones included in this investigation without syndromic hearing impairment (e.g., Usher syndrome), the sensory deficit is purely visual in nature, thus compromising behavioral identification of AV stimuli. In today’s society, our interaction with the environment and others have become far more visual than auditory (e.g., most people age 45 or below nowadays communicate via text and social media than by phone call). Thus, the fact that the behavioral identification of auditory (A) tokens by IRD patients was not proportionally lower as seen in controls suggests that, in controls, visual cues are cognitively the ones relied upon the most, whereas IRD subjects, being visually impaired, compensate proportionally with higher sensitization to their auditory function (e.g., Fig. 4b). Nevertheless, the slower RTs for IRD subjects in these conditions suggest this enhanced auditory sensitivity is at the expense of slower processing, presumably reflecting a compensatory strategy from the impaired visual input.

Analysis of EEG frequency power revealed increased spectral slopes (Fig. 5b) in the IRD group relative to controls for simultaneous A and V stimulation and singular V stimulation. Increase in EEG slope could reflect higher lower frequency energy in A and decreased higher frequency energy for V, consistent with the interpretation of low and high bands as network communication and stimulus coding, respectively. In brief, we found that larger (more negative) slopes are associated with poorer AV perception (Fig. 5c). If auditory responses are dominated by increased alpha activity in IRDs relative to controls, this effect may point to an enhancement in auditory sensitivity of IRD subjects, as suggested by the steeper EEG spectral slopes (Fig. 5b) and larger ERPs as we find in auditory-only conditions (Fig. 4). Interestingly, spectral and temporal enhancements in IRD’s brain responses were observed in the absence of AV integration (Fig. 4c). Thus, an alternate account of the observed IRD auditory enhancements may be that long-term visual deprivation causes higher-level cortical disinhibition that produces broad activation and permits enhanced neural encoding of sound. Indeed, complementary studies have suggested that other forms of sensory loss/deprivation (e.g., hearing loss) can alter cortical response activity due to a disinhibition of sensory coding ([13]b).

Our study aimed to identify changes in cortical processing of multimodal stimuli following long term visual loss. For IRD subjects, we observed that auditory inputs engage more responsiveness from the cortex and seem to entrain neural activity at lower frequency bands of the EEG, particularly at scalp locations over temporal and parietal-occipital junctions. Larger responsivity in these areas could reflect changes in neural resources mediating task execution. For example, it is conceivable that given their partial sensory deficit, IRD individuals required higher levels of attentional deployment or cognitive processing to arrive at their behavioral judgments. This notion is supported by our behavioral RT data, which showed much longer response times in IRDs relative to controls when identifying the perceived motion of AV stimuli (Fig. 3b). As stated by Smilek et al. [44] and Myles et al. [35], higher-level brain regions may collect information transmitted from various sensory cortices, and project this information to the brain areas eliciting the concurrent perception. It is conceivable this information routing is still possible but simply more sluggish in IRD. This proposition is evident by the chance behavioral accuracy at the expense of slower response times exhibited by IRD subjects (Fig. 3).

Several frameworks have been developed to describe how brain networks manage simultaneous sensory inputs. The *disinhibited feedback model* suggests that multi-sensory input results in disinhibited feedback from higher-level cortical areas in the processing hierarchy [1, 22]. This would imply that, for AV stimulation, higher-level cortical areas collect information transmitted from the sensory cortex, and project this information to the brain areas eliciting the concurrent percept. Previous studies have demonstrated that areas of the posterior inferior temporal cortex, the parieto-occipital junction, and V4 were activated during word listening more than during tone listening in individuals with congenital cross-sensory integration deficits [35, 44]. In this regard, a specific sensory deficit in one modality may induce a form of cortical remapping and recruit compensatory processing in brain areas not associated with the impairment (e.g., recruitment of auditory cortex in blind individuals; [17]).

Multisensory processing can be directly assessed using audiovisual AV looming and receding stimuli, which convey the sense of motion [14]. For these stimuli, the integration of A and V looming signals may be mediated by functional interactions between primary auditory cortex and the superior temporal sulcus (STS), two areas involved in integrating behaviorally relevant AV signals [3, 19, 31]. AV looming signals also elicit increased gamma-band coherence between these areas, relative to unimodal (A or V stimulation) or receding motion signals [31]. Increased neuronal coherence might result in more efficient communication between these areas and fronto-parietal networks [21, 42, 47], resulting in better-coordinated responses to looming (i.e., approaching) events [2, 43]. The STS is known to be involved in the perception of biological motion, which may explain why impending signals seem to heavily recruit this area and reveal the largest group differences in our neural data (Fig. 5c). Germane to the current study, AV processing is not static, but has been shown to vary with certain experiential factors (e.g., musical training) and learning [8, 38]. While AV processing can be positively enhanced with learning and experience presumably, we show here that it can be negatively altered in cases of visual sensory deficit.

Limitations of this study are worth noting. While group differences in the EEG between IRDs and controls (pooled across conditions in Fig. 6) showed a large effect size (d = 2.07; α = 0.05, power = 98%, two–tailed *t*-test), our limited sample of different IRD subgroups (i.e., Fig. 7) limits our conclusions regarding possible differential effects in AV processing between RP, BBS, and CORD pathologies. Indeed, comparisons between the RP and CORD spectral slopes in the V condition (which shows the largest subgroup differences in Fig. 7) achieves an effect size of d = 0.81. However, this result is tempered by the fact that the corresponding power is only 7%. [*n* = 50 subjects would be needed to detect an effect at 80% power]. Additional studies on larger population samples, especially by disease subgroup, are needed to confirm the different trends noted here in handling looming vs. receding stimuli depending on the vision loss being peripheral (RP) vs. central (CORD), and in subjects with Bardet-Biedl syndrome (BBS). It is possible that comparing different stratifications of IRD may prove difficult given heterogeneity between the populations. Nevertheless, we demonstrate that EEG responses can reveal robust differences between IRD patients and normal controls. We anticipate that the larger study population planned for in this study would not only reinforce our initial findings, but clearly delineate which subtype produced the highest degree of auditory compensation.

## Conclusion

Neural activity in IRD subjects offer insight into how auditory and visual sensory inputs are processed when the visual input is diminished over time. We found evidence for enhanced (or at least more sensitized) auditory neural encoding in participants with IRDs, which may relate to compensatory recruitment of auditory system function with gradual visual loss and increased sensitization, or differential reweighting of the senses, towards sound processing. Our approach utilized salient multisensory stimuli that revealed compensatory neuroplasticity in the brain processing of IRD patients. More broadly, this approach could be applied in a rehabilitation/training paradigm to further enhance auditory sensitivity in cases of progressive visual loss in attempts to enhance function in one sensory area as the input to another modality diminishes.

## Additional file

Additional file 1: Figure S1. IRD patient selection criteria. (DOCX 761 kb)

## Abbreviations

A + V: Summed unimodal
A: Auditory
AV: Audiovisual
BBS: Bardet-biedl syndrome
CORD: Cone-rod dystrophy
EEG: Electroencephalography
ERG: Electroretinogram
ERP: Event-related brain potentials
FAF: Fundus Autofluorescence
fMRI: Functional magnetic resonance imagery
IRDs: Inherited retinal dystrophies
PCA: Principal component analysis
PSD: Power spectral density
RP: Retinitis pigmentosa
RT: Reaction times
SD-OCT: Spectral domain optical coherence tomography
STS: Superior temporal sulcus
V: Visual
