# Supplementary material for: A pilot investigation of audiovisual processing and multisensory integration in patients with inherited retinal dystrophies
Source: BMC Ophthalmol. 2017 Dec 7;17:240. doi: 10.1186/s12886-017-0640-y (PMC5719743; doi:10.1186/s12886-017-0640-y)
Supplement: Additional file 1: Figure S1. — IRD patient selection criteria. (DOCX 761 kb) [file 12886_2017_640_MOESM1_ESM.docx]

**
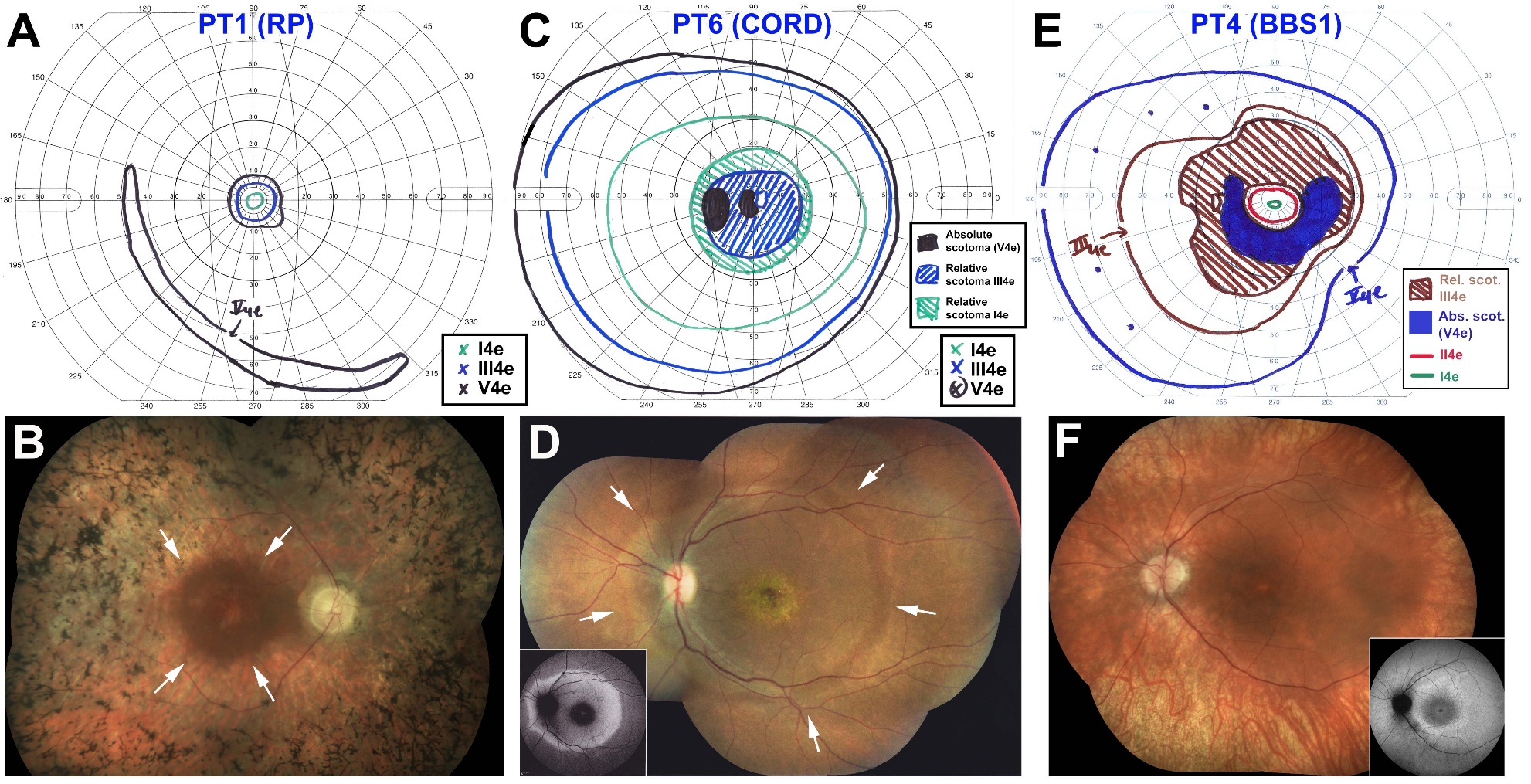
*Additional file 1***

**Figure S1. Examples of visual field and disease patterns included in this study. A.** **PT1**, Example of typical Goldmann visual fields (GVFs) in RP (peripheral field loss). **B.** Note the small central GVF residue in RP matching closely the area of best RPE/retina preservation (white arrows) visible ophthalmoscopically. **C. PT6,** Example of **typical** GVF in CORD (central scotoma of variable density). Note how the density of the scotoma matches closely the area of metallic reflexes seen in **D** (white arrows) – the inset shows a fundus autofluorescence (FAF) image from the same case, further emphasizing the close match between GVF function loss and the hyper-AF area. **E, PT4**, Example of GVF in early to mid- stage BBS1 with an essentially full field to the V4e target, a ring scotoma (mostly relative in nature), and a tiny area of central I4e detection. **F.** The fundus of PT4 is relatively modest in clinical findings, but the FAF image (inset) shows a pericentral ring of hyper-AF and an additional internal (paracentral) ring of hypo-AF, matching closely the edges of the denser part of the ring scotoma and the gap in sensitivity between the III4e target and the I4e target on GVF testing, respectively.
